# Randomized, Placebo-Controlled Trial of Transplacental Antibiotic Prophylaxis of Neonatal Group B Streptococcal Colonization and Bacteremia in Rabbits

**DOI:** 10.1155/S1064744997000628

**Published:** 1997

**Authors:** Arda Lembet, Robert S. McDuffie, Ronald S. Gibbs

**Affiliations:** 1 Department of Obstetrics and Gynecology University of Colorado Health Sciences Center Denver CO USA; 2 Department of Obstetrics and Gynecology Kaiser Permanente Franklin 2045 Franklin Street Denver CO 80205 USA

## Abstract

*Objective:* We evaluated the effect of maternal administration of ampicillin/sulbactam on colonization and bacteremia in newborn rabbits after intracervical inoculation of mothers with group B streptococci (GBS).

*Methods:* New Zealand white rabbits on day 30 of a 31-day gestation were inoculated intracervically with 10^4^−10^5^ colony forming units (cfu) GBS. Two hours after inoculation mothers received ampicillin/sulbactam (50 mg/kg) or saline (control) intramuscularly as a single dose, in a randomized double-blinded manner. We induced labor 4 h later with intramuscular oxytocin. At delivery, cultures for GBS were taken from neonatal oropharynx. Thereafter, cultures were taken from neonatal oropharynx and anorectum daily and from neonatal heart at death or after 96 h. Sample size analysis showed a need for 17 pups in each group.

*Results:* In the control group, induction failed in one animal that was excluded from analysis. At birth, 0 of 39 pups of treated does had positive oropharyngeal cultures compared to 26 of 27 (96%) pups of saline-treated does (*P* < 0.0001). Pups treated with antibiotic in utero were also significantly less likely to have positive oropharyngeal cultures at 24, 48, and 72 h after birth compared to controls (24 h, 0% vs. 100%, *P* < 0.0001; 48 h, 8% vs. 100%, *P* < 0.0001; 72 h, 16% vs. 100%, *P* < 0.0001). Treated pups were significantly less likely to have positive anorectal cultures at 24, 48, and 72 h after birth compared to control animals (24 h, 0% vs. 100%, *P* < 0.0001; 48 h, 0% vs. 95%, *P* < 0.0001; 72 h, 0% vs. 92%, *P* < 0.0001). Treated pups were significantly less likely to have positive heart cultures at 72 h after birth compared to controls (11% vs. 92%, *P* < 0.0002). Cumulative neonatal survival was higher in treated pups compared to controls at 72 and 96 h after birth (72 h, 32% vs. 0%, *P* = 0.0003; 96 h, 26% vs. 0%, *P* = 0.015).

*Conclusions:* Single dose transplacental prophylaxis given 4 h before delivery resulted in decreased neonatal GBS colonization and bacteremia and improved neonatal survival in rabbits.


Infectious Diseases in Obstetrics and Gynecology 5:355–358 (1997)
© 1998 Wiley-Liss, Inc.

### Randomized, Placebo-Controlled Trial of Transplacental Antibiotic Prophylaxis of Neonatal Group B Streptococcal Colonization and Bacteremia in Rabbits

Arda Lembet,[1] Robert S. McDuffie, Jr.,[2]* and Ronald S. Gibbs[1]
[1]*Department of Obstetrics and Gynecology, University of Colorado Health Sciences Center, Denver, CO*
[2]*Department of Obstetrics and Gynecology, Kaiser Permanente Franklin, Denver, CO*

ABSTRACT

*Objective:* We evaluated the effect of maternal administration of ampicillin/sulbactam on colonization and bacteremia in newborn rabbits after intracervical inoculation of mothers with group B streptococci (GBS).

*Methods:* New Zealand white rabbits on day 30 of a 31-day gestation were inoculated intracervically with $10^4$–$10^5$ colony forming units (cfu) GBS. Two hours after inoculation mothers received ampicillin/sulbactam (50 mg/kg) or saline (control) intramuscularly as a single dose, in a randomized double-blinded manner. We induced labor 4 h later with intramuscular oxytocin. At delivery, cultures for GBS were taken from neonatal oropharynx. Thereafter, cultures were taken from neonatal oropharynx and anorectum daily and from neonatal heart at death or after 96 h. Sample size analysis showed a need for 17 pups in each group.

*Results:* In the control group, induction failed in one animal that was excluded from analysis. At birth, 0 of 39 pups of treated does had positive oropharyngeal cultures compared to 26 of 27 (96%) pups of saline-treated does ($P < 0.0001$). Pups treated with antibiotic in utero were also significantly less likely to have positive oropharyngeal cultures at 24, 48, and 72 h after birth compared to controls (24 h, 0% vs. 100%, $P < 0.0001$; 48 h, 8% vs. 100%, $P < 0.0001$; 72 h, 16% vs. 100%, $P < 0.0001$). Treated pups were significantly less likely to have positive anorectal cultures at 24, 48, and 72 h after birth compared to control animals (24 h, 0% vs. 100%, $P < 0.0001$; 48 h, 0% vs. 95%, $P < 0.0001$; 72 h, 0% vs. 92%, $P < 0.0001$). Treated pups were significantly less likely to have positive heart cultures at 72 h after birth compared to controls (11% vs. 92%, $P < 0.0002$). Cumulative neonatal survival was higher in treated pups compared to controls at 72 and 96 h after birth (72 h, 32% vs. 0%, $P = 0.0003$; 96 h, 26% vs. 0%, $P = 0.015$).

*Conclusions:* Single dose transplacental prophylaxis given 4 h before delivery resulted in decreased neonatal GBS colonization and bacteremia and improved neonatal survival in rabbits. Infect. Dis. Obstet. Gynecol. 5:355–358, 1997. © 1998 Wiley-Liss, Inc.

KEY WORDS
group B streptococcus; rabbit; animal model; neonatal colonization; neonatal sepsis

In 1996, the Centers for Disease Control (CDC)[1] and the American College of Obstetricians and Gynecologists (ACOG)[2] published recommendations for prevention of early onset neonatal group B streptococcal (GBS) sepsis by intrapartum chemoprophylaxis. Although several clinical trials in hu-

Contract grant sponsor: Pfizer-Roerig, Incorporated.
*Correspondence to: Dr. Robert S. McDuffie, Jr., Department of Obstetrics and Gynecology, Kaiser Permanente Franklin, 2045 Franklin Street, Denver, CO 80205.

Received 8 July 1997

Accepted 27 October 1997

mans have demonstrated that the use of intrapartum prophylaxis can reduce cases of sepsis in infants,[3–8] only one trial showed a statistically significant reduction, and none used a randomized, placebo-controlled experimental design. In a meta-analysis, Ohlsson and Myhr[9] argued that the effectiveness of intrapartum prophylaxis has not been rigorously shown.

We believe that our rabbit model of ascending infection in pregnancy may provide important information relevant to the pathogenesis and prevention of GBS sepsis in human newborns. In past work, we have demonstrated that inoculation of the rabbit cervix with GBS at 70% gestation led to high rates of clinical intra-amniotic infection in untreated animals.[10] Recently, we treated pregnant rabbits with antibiotic or saline, induced labor, and inoculated the oropharynx of each pup with GBS. Pups born to antibiotic-treated mothers had significantly decreased neonatal colonization and bacteremia[11] compared to pups born to saline-treated mothers. In the present work, we have sought to more closely mimic vertical transmission in humans by inoculating the cervix of the mother prior to delivery. We have tested the hypothesis that a single dose of antibiotic administered prior to labor reduces neonatal GBS colonization and sepsis.

### SUBJECTS AND METHODS

This protocol was approved by the Animal Use and Care Committee at the University of Colorado Health Sciences Center. We used timed pregnant New Zealand white rabbits with a 31-day gestation. We obtained these animals from approved vendors (Myrtle's Rabbitry, Thompson Station, TN).

On day 30 (97% of gestation), the maternal cervices were inoculated with $10^4$–$10^5$ colony forming units (cfn) type Ia GBS endoscopically with a technique previously described.[10] In previous experiments, this inoculum size has led to clinical intra-amniotic infection. In brief, animals were anesthetized and an endoscope was placed in the vagina and advanced to the cervix. Under direct visualization, a cannula was advanced through the operating port into the cervix, and a bacterial inoculum was given. The animals were then returned to their cages.

Two hours after inoculation, mothers received either 50 mg/kg ampicillin/sulbactam[11] or sterile saline intramuscularly in a blinded manner. This dose of ampicillin for a 4 kg rabbit would have been equal to 2.6 g in a 70 kg human. A schedule of group assignments was generated from a table of random numbers. Two animals were inoculated each time the experiment was performed. Syringes containing antibiotic or saline were wrapped with tape to prevent the investigators from knowing the color of the solution. These syringes were labeled with the rabbit's experimental number. Four hours after treatment (6 h after inoculation), labor was induced with oxytocin intramuscularly. Delivery usually occurred within 30 min. At delivery, viability was assessed, and cultures for GBS were taken from the pups' oropharynges. Thereafter, pups were observed on a daily basis for up to 96 h. Syringe feeding was necessary for most pups because of rejection by the mother.

Swabs for culture were collected from the oropharynx and anorectum of each pup daily and plated onto 5% sheep blood agar or into selective broth media (Todd-Hewitt broth containing nalidixic acid and gentamicin) with subculture onto 5% sheep blood agar. Blood for culture was taken from the neonatal heart at death or after 96 h. GBS were identified by Gram stain, catalase test, and the Christie-Atkins-Munch-Petersen (CAMP) test. Outcomes measured were the rates of 1) positive culture for GBS at each site (oropharynx, anorectum, and heart) for each 24-h period, and 2) cumulative neonatal survival for each 24-h period. We used Fisher's exact test and chi-square analysis to compare rates. We considered $P < 0.05$ to be significant.

For sample size analysis, we assumed that alpha = 0.05, beta = 0.2, the proportion of positive oropharyngeal cultures in the placebo group of 90% at 24 h after delivery, and a difference in proportion between antibiotic and placebo groups of 50%. With these assumptions, a total of 17 pups were required in each group. Since the litter size varied from 4 to 8, we estimated that a total of 5 mothers in each group would be required.

### RESULTS

Five does were randomized to each group. In the control group, induction of labor failed in one animal that was excluded from analysis. Table 1 shows the positive neonatal oropharyngeal cultures by time from delivery and treatment group. From birth through 72 h, pups born to antibiotic-treated

TABLE 1. Positive neonatal oropharyngeal cultures by time from delivery and treatment group

| Time | Ampicillin/sulbactam | Saline | *P* |
|---|---|---|---|
| Birth | 0/39 | 26/27 (96%) | <0.0001 |
| 24 h | 0/31 | 22/22 (100%) | <0.0001 |
| 48 h | 2/26 (8%) | 19/19 (100%) | <0.0001 |
| 72 h | 3/19 (16%) | 13/13 (100%) | <0.001 |
| 96 h | 0/10 | 0/0 | NA[a] |

[a]NA = not applicable.

mothers had significantly fewer positive cultures than pups born to controls. We obtained similar results for neonatal anorectal cultures at 24, 48, and 72 h after birth (Table 2). Compared to the control group, the treated pups had less bacteremia at 24, 48, and 72 h with statistical significance achieved at 72 h (Table 3). Because all of the pups in the control group died by 72 h, no results are available at 96 h in Tables 1–3. Table 4 shows the cumulative neonatal survival by time from delivery and treatment group. At both 72 and 96 h after delivery, significantly more pups survived in the treated group compared to the control group.

TABLE 2. Positive neonatal anorectal cultures by time from delivery and treatment group

| Time | Ampicillin/sulbactam | Saline | *P* |
|---|---|---|---|
| 24 h | 0/31 | 22/22 (100%) | <0.0001 |
| 48 h | 0/26 | 18/19 (95%) | <0.0001 |
| 72 h | 0/19 | 12/13 (92%) | <0.0001 |
| 96 h | 0/10 | 0/0 | NA[a] |

[a]NA = not applicable.

TABLE 3. Positive neonatal heart cultures by time from delivery and treatment group

| Time | Ampicillin/sulbactam | Saline | *P* |
|---|---|---|---|
| 24 h | 0/2 | 3/3 (100%) | NA[a] |
| 48 h | 3/10 (30%) | 5/6 (83%) | 0.12 |
| 72 h | 1/9 (11%) | 12/13 (92%) | 0.002 |
| 96 h | 0/10 | 0/0 | NA |

[a]NA = not applicable.

TABLE 4. Cumulative neonatal survival by time from delivery and treatment group

| Time | Ampicillin/sulbactam (N = 31) | Saline (N = 22) | *P* |
|---|---|---|---|
| 24 h | 29 (94%) | 19 (86%) | NS[a] |
| 48 h | 19 (61%) | 13 (59%) | NS |
| 72 h | 10 (32%) | 0 | 0.003 |
| 96 h | 8 (26%) | 0 | 0.015 |

[a]NS = not significant.

### DISCUSSION

This experiment provides the first blinded, placebo-controlled evidence, in any species, that maternal chemoprophylaxis prevents vertical transmission of GBS and neonatal colonization and sepsis. A single intramuscular dose of ampicillin/sulbactam, given 4 h before delivery, dramatically improved outcomes in this model. We demonstrated significantly lower rates of neonatal colonization of the oropharynx not only at birth but also within 72 h of birth. We found lower rates of positive neonatal heart cultures in the antibiotic-treated group at 48 and 72 h after birth. Our highest rates of neonatal survival in the treated group at 72 and 96 h after birth correlated with the timing of the lowest rates of positive neonatal heart cultures.

These striking results in the rabbit are subject to several limitations when compared to women. First, we administered the antibiotic intramuscularly rather than intravenously as in humans. Second, we knew the exact time of the beginning of infection. Third, we studied only one time interval from antibiotic administration to delivery, namely 4 h. We cannot comment on whether a shorter or longer interval would produce equivalent results. In humans, bactericidal concentrations of ampicillin in the fetal serum and amniotic fluid have been documented as soon as 5 min after maternal ampicillin injection.[12] We believe that the ampicillin portion of ampicillin/sulbactam resulted in the clinical effect seen. Although all treated pups had negative oropharyngeal cultures at birth and at 24 h, some pups later developed positive oropharyngeal and heart cultures. Possible explanations include a failure of the culture to detect low-level colonization initially present or cross-contamination within a litter. Further, our experimental protocol did not provide continued antibiotic treatment after delivery as advocated in some human situations by the CDC and ACOG guidelines. In the current work, 21 (68%) pups in the treated group died by 72 h after inoculation. Although some of these deaths were associated with GBS bacteremia (4/21, 19%), most were the result of the interruption of nesting behavior. Nevertheless, the results clearly show significantly fewer deaths with bacteremia in the treated group. Finally, in the untreated group, early neonatal mortality was 100%. In humans, although vertical transmission of GBS from mothers to infants is 50–70%, invasive GBS disease occurs in only 1% of colonized neonates.

We believe these experiments represent an important advance in our work on the pathogenesis of GBS disease in this animal model. In early experiments, we demonstrated that intracervical inoculation of GBS into rabbits at 70% gestation led to fulminant intra-amniotic infection in all cases.[10] We attempted to establish a model of chronic vaginal carriage of GBS similar to humans but found that vaginal inoculation led to clinical and microbiologic evidence of infection in nearly 50% of cases.[13] To study rates of neonatal GBS colonization and sepsis after maternal antibiotic administration, we then inoculated pups orally after induction of labor. We found that a single dose of antibiotic administered to the mother within 2–6 h of birth led to lower rates of neonatal colonization and sepsis compared to a control group.[11] In the present work, the experimental time sequence of inoculation, treatment, and delivery more closely mimics the situation in humans.

Despite the obvious interspecies differences, we believe that this model demonstrates some important features of neonatal GBS disease in humans. First, vertical transmission led to a high neonatal colonization rate as in humans. Second, colonization led to invasive disease and neonatal death. Third, a single dose of antibiotic prophylaxis 4 h prior to delivery was effective in lowering colonization rates and death with bacteremia. We conclude that the results of these experiments provide the first blinded, placebo-controlled evidence that intrapartum chemoprophylaxis reduces the rate of GBS sepsis in neonates.

### REFERENCES

1. Centers for Disease Control and Prevention: Prevention of perinatal group B streptococcal disease: A public health perspective. MMWR 46(RR-7):1–24, 1996.
2. American College of Obstetricians and Gynecologists: Prevention of early-onset group B streptococcal disease in newborns. American College of Obstetricians and Gynecologists Committee Opinion, June 1996.
3. Allardice JG, Baskett TF, Seshia MM, Bowman N, Malazdrowicz R: Perinatal group B streptococcal colonization and infection. Am J Obstet Gynecol 142:617–620, 1982.
4. Boyer KM, Gotoff SP: Prevention of early-onset neonatal group B streptococcal disease with selective intrapartum chemoprophylaxis. N Engl J Med 314:1665–1669, 1986.
5. Morales WJ, Lim DV, Walsh AF: Prevention of neonatal group B streptococcal sepsis by the use of a rapid screening test and selective intrapartum chemoprophylaxis. Am J Obstet Gynecol 155:979–983, 1987.
6. Morales WJ, Lim D: Reduction of group B streptococcal maternal and neonatal infections in preterm pregnancies with premature rupture of membranes through a rapid identification test. Am J Obstet Gynecol 157:13–16, 1987.
7. Tuppurainen N, Hallman M: Prevention of neonatal group B streptococcal disease: Intrapartum detection and chemoprophylaxis of heavily colonized parturients. Obstet Gynecol 73:583–587, 1989.
8. Mattorras R, Garcia-Perez A, Omenaca F, Diez-Enciso M, Madero R, Usandizaga JA: Intrapartum chemoprophylaxis of early-onset group B streptococcal disease. Eur J Obstet Gynecol Reprod Biol 40:57–62, 1991.
9. Ohlsson A, Myhr TL: Intrapartum chemoprophylaxis of perinatal group B streptococcal infections: A critical review of randomized controlled trials. Am J Obstet Gynecol 170:910–917, 1994.
10. McDuffie R, Gibbs R: Animal models of ascending genital-tract infection in pregnancy. Infect Dis Obstet Gynecol 2:60–70, 1994.
11. McDuffie R, Blanton S, Charland S, Gibbs R: The effect of timing of single-dose transplacental ampicillin-sulbactam therapy for prevention of neonatal group B streptococcal colonization and bacteremia in a rabbit model. Am J Obstet Gynecol 175:406–410, 1996.
12. Bloom S, Cox S, Bawdon R, Gilstrap L: Ampicillin for neonatal group B streptococcal prophylaxis: How rapidly can bactericidal concentrations be achieved? Am J Obstet Gynecol 175:974–976, 1996.
13. McDuffie R, Gibbs R: Ascending group B streptococcal genital infection in the rabbit model. Am J Obstet Gynecol 175:402–405, 1996.
